# Examining the Suitability of NetFlow Features in Detecting IoT Network Intrusions

**DOI:** 10.3390/s22166164

**Published:** 2022-08-17

**Authors:** Mohammed Awad, Salam Fraihat, Khouloud Salameh, Aneesa Al Redhaei

**Affiliations:** 1 Department of Computer Science and Engineering, American University of Ras Al Khaimah, Ras Al Khaimah P.O. Box 72603, United Arab Emirates; 2 Artificial Intelligence Research Center (AIRC), College of Engineering and Information Technology, Ajman University, Ajman P.O. Box 346, United Arab Emirates

**Keywords:** Internet of Things, cyber security, Network Intrusion Detection System, machine learning, feature selection

## Abstract

The past few years have witnessed a substantial increase in cyberattacks on Internet of Things (IoT) devices and their networks. Such attacks pose a significant threat to organizational security and user privacy. Utilizing Machine Learning (ML) in Intrusion Detection Systems (NIDS) has proven advantageous in countering novel zero-day attacks. However, the performance of such systems relies on several factors, one of which is prediction time. Processing speed in anomaly-based NIDS depends on a few elements, including the number of features fed to the ML model. NetFlow, a networking industry-standard protocol, offers many features that can be used to predict malicious attacks accurately. This paper examines NetFlow features and assesses their suitability in classifying network traffic. Our paper presents a model that detects attacks with (98–100%) accuracy using as few as 13 features. This study was conducted using a large dataset of over 16 million records released in 2021.

## 1. Introduction

During the last few decades, technological advancements have given rise to several innovative concepts, such as the Internet of Things (IoT), which played an increasingly important role in a variety of areas, such as smart cities, healthcare, and education. The IoT is a new technology paradigm envisioned as an ecosystem of interconnected ”things” aiming to bring every physical device into the digital network [1]. Connecting billions of devices through sensors, actuators, and other components, IoT is anticipated to have 75 billion machine-to-machine connections by 2025 and is expected to generate 79.4 zettabytes of data [2] With the tremendous amount of data flowing between IoT devices and across networks, protecting these networks against IoT breaches becomes a high priority. The world is experiencing a significant increase in IoT cyberattacks, which increased by 100% in 2021, according to Kaspersky, a leading anti-virus company. The low memory and cost of IoT devices make them even more challenging to secure [3]. Among recent alarming incidents of IoT attacks is the Verkada breach [4], in March 2021, where a group of hackers managed to access and control thousands of Verkada surveillance cameras. In addition, they could access video recordings stored in the cloud of more than 24,000 clients and utilize the cameras to carry out future attacks. Another intense incident occurred in July 2021 [5] when My Book Live and My Book Live Duo devices’ storage was completely erased. This happened during a cyberattack against Western Digital, when hackers were able to remotely perform a factory reset without having a password due to a critical security vulnerability. In all of these incidents, IoT devices were targeted by several malicious attacks such as distributed denial of service (DDoS) attacks [6], man-in-the-middle attacks [7], spoofing attacks [8], targeted code injection [9], and other unprecedented types of attacks. Thus, there has been an increased concern about enhancing the effectiveness of the current Network Intrusion Detection Systems (NIDS) to detect new attacks [10].

Network Intrusion Detection Systems (NIDS) [11] are security tools that monitor network traffic flow to detect IoT attacks. They are designed to enhance the security of information and communication systems. NIDS can be categorized into signature-based [12] and anomaly-based [13]. The signature-based NIDS compares the incoming traffic to a database of known attacks based on the signatures. However, in the anomaly-based approach, a normal profile is created based on the normal behavior of the network, and any deviation from this is viewed as an attack. In other words, The signature-based method compares network traffic against a pre-existing list of compromises to detect a known intrusion efficiently. On the other hand, the anomaly-based method relies on machine learning to identify a threat making it ideal for spotting unknown attacks. However, like any system that relies on ML, actual accuracy may vary. Understandably, the pros of one approach are the cons of the other. Thus, some IDS combine signature and anomaly-based approaches to benefit from both.

Security threats are continuously evolving. Thus, NIDS detection models that rely on old datasets have clear limitations. Our paper utilizes a recently published (2021) large dataset with around 17 million data rows known as NF-ToN-IoT-v2 [14]. While the dataset’s existing features provide outstanding accuracy, we are interested in conducting binary- and multi-class classification using a subset of the dataset’s 43 features. Reducing the number of utilized features can minimize the prediction time and necessary storage and enhance networks’ operational functionality. However, it will also reduce prediction accuracy. Thus, our focus was on investigating the right balance. Our results were acquired using the entire dataset without any sampling.

As mentioned earlier, our dataset of choice, NF-ToN-IoT-v2, is an IoT dataset consisting of 43 features with a total of 16,940,496 data rows. Each data row is classified as an attack or benign. The attacks make up 63.99% of the dataset, while the benign samples represent 36.01%. Furthermore, the dataset contains nine different types of attacks, namely Backdoor, Denial of Service (DoS), Distributed Denial of Service (DDoS), Injection, Man in the Middle (MITM), Password, Ransomware, Scanning, and Cross-site Scripting (XSS) [14]. Such attacks compromise the IoT system’s security by violating one or more of its CIA principles [15] (Confidentiality, Integrity, and Availability).

Table 1 below shows the distribution of NF-ToN-IoT-v2 (16,940,496) data rows over the nine attacks and the benign flow (ten classes in total) [14]. The dataset consists of 43 features, as shown in Table 2 [16]. More details on NF-ToN-IoT-v2 origins, Netflow features, previous versions, and utilizations are presented in Section 2.

In this paper, we used this dataset to efficiently classify network flows into benign and attacks (binary-classification) and to predict the exact type of attack (multi-classification). Our research target was to achieve these tasks with high accuracy while utilizing a lower number of features. In our assessment, we applied four shallow machine learning classifiers, namely Decision Trees (DT), Random Forest (RF), XGBoost (XGB), and Naïve Bayes (NB).

The paper contributes to the literature by utilizing a fairly large, recently published dataset (2021). The dataset has been fully utilized without any sampling. While other research has achieved high accuracy using an extensive set of features, our aim was to reduce the number of features while still achieving a highly accurate detection system. A lower number of features results in a faster prediction time and lower storage space, which translates into a better operational system.

The rest of the paper is divided as follows: Section 2 reviews the literature; Section 3 presents the methodology; Section 4 explains how the features were chosen; Section 5 covers the results and their analysis; Section 6 concludes the paper.

## 2. Related Work

Our work is based on a dataset created by Sarhan et al. [17]. Thus, we will start this section by summarizing their highly influential work. In their Intrusion Detection Work, Sarhan et al. [14,18,19] have utilized and created several datasets with network traffic data. Sarhan et al. have been interested in utilizing Netflow features to standardize NIDS datasets [14,18]. NetFlow is a Cisco standard that collects traffic data as it flows across the network [18]. The features extracted from Netflow have proven valuable in detecting network attack [14]. In their early work, Sarhan et al., used pcap files of existing datasets to generate several datasets with a few Netflow features (up to eight features) [18]. For example, NF-ToN-IoT was created using the publicly available ToN-IoT dataset [20]. The binary-classification results of NF-ToN-IoT showed a slight improvement over ToN-IoT with an F1-score of 1.00 compared to 0.99 in the original dataset [18]. However, the multi-classification F1-score weighted average dropped from 0.87 to 0.60. Both datasets contain 10 classes. Extra Trees ensemble classifier was used to assess both datasets. In later work, Sarhan et al. expanded the utilized Netflow features to 43 and generated several datasets, including NF-ToN-IoT-v2 [14]. The use of 43 features made a significant improvement in comparison to the previous two versions. For example, the second version’s F1-score was 1.00 in binary-classification and the multi-classification F1-score weighted average was 0.98.

In addition to the dataset creators, Le et al. proposed a binary and multi-class detection model using 20 features from NF-ToN-IoT-v2 [21]. The authors reported an F-1 score of 1.00 and an area under the curve (AUC) value of 93%. A higher AUC means a better distinction between the classes. However, we argue that our approach achieved similar results using fewer features (13 and 17). Furthermore, our proposed method resulted in an AUC of 97.7%.

In another paper, Sarhan et al. [22] compared between original, Netflow feature based (43 features), and CICFlowMeter feature-based (83 features) datasets. CICFlowMeter is also a network flow traffic generator. The study concluded that the Netflow-based features dataset achieved higher accuracy in lower prediction time.

Furthermore, in another recent study, Sarhan et al. analyzed the classification performance of three datasets using several ML models and feature extraction algorithms and concluded that no ML model and feature extraction algorithm combination work best across all datasets [19]. Such a conclusion motivated our work and interest in researching a trade-off between accuracy and performance.

Dias et al. [23] utilized Netflow data to detect attacks. In their work, they used 12 fixed Netflow features in addition to a set of dynamically defined and extracted features from the network data. They experimented with 52 to 412 features using CIC-IDS-2018 and a confidential military dataset. They achieved an F1-score of 0.97.

In another work, Liu et al. [24] converted a 2013 one-dimensional Netflow dataset into two-dimensional images and fed it to a Convolutional Neural Network (CNN). The authors reported an accuracy of 95.86%. However, they noted that the additional computational time to convert the data into images made it impractical in real-life scenarios.

Other works that utilized Netflow in intrusion detection include [25,26]. In [25], Krishnamurthy et al. proposed a framework that predicts attacks using Netflow logs using machine learning. The purpose of their framework is to provide analysts with a readable explanation of the logic behind the classification. In [26], Haghighat et al. applied deep learning using 92 Netflow extracted features. In their work, they utilized a dataset from 2011 called CTU 13 and reported an accuracy above 99%.

Further research in the domain is summarized below with the purpose of highlighting the methods adopted. It is difficult to compare against the accuracy and speed of models that were tested on different datasets using machines with different specifications.

In [27], Abu Al-Haija and Al-Badawi implemented several ML models (ensemble, neural, and kernel) and assessed their ability to anomaly detect intrusions on IoT networks. The designed NIDS was tested on two datasets, namely distilled-Kitsune-2018 and Network Security Laboratory-Knowledge Discovery Databases (NSL-KDD). Each dataset consisted of around 150,000 network traffic records. The authors concluded that ensemble methods achieve the highest accuracy while neural network methods had the highest prediction speed. In an earlier work, Abu Al-Haija and Saleh achieved an accuracy of 98.2% in classifying IoT cyber-attacks using the Convolutional Neural Network model over the NSL-KDD dataset [28].

In [29], Verma and Ranga studied a system specific to detecting DoS attacks. The authors assessed the accuracy and performance of several models using CIDDS-001, UNSW-NB15, and NSL-KDD datasets. The study concluded that both Classification and Regression trees (CART) and XGBoost are the most practical classifiers in terms of performance and prediction speed.

In [30], an analysis of NIDS classification performance on a benchmark data set, ISCX 2012, was performed using the SVM classification algorithm. In addition, two major methods for selecting features are examined: Recursive Feature Elimination (RFE) and Recursive Feature Addition (RFA). In their study, the authors observed that RFE performs better when it comes to independent features, while RFA works better when tackling interdependent features.

In [31], the authors used several feature selection algorithms such as genetic algorithm, particle swarm optimization, firefly optimization, and gray wolf optimization to analyze the performance of NIDS. Their model was evaluated using support vector machines (SVMs) and machine learning classifiers. The UNSW-NB15 dataset is used as input for the experiment. The authors concluded that a system with fewer features would be more accurate.

## 3. Methodology

The section below presents our research methodology, starting with an illustration of the proposed system’s architecture.

### 3.1. Architecture

**Figure 1:**
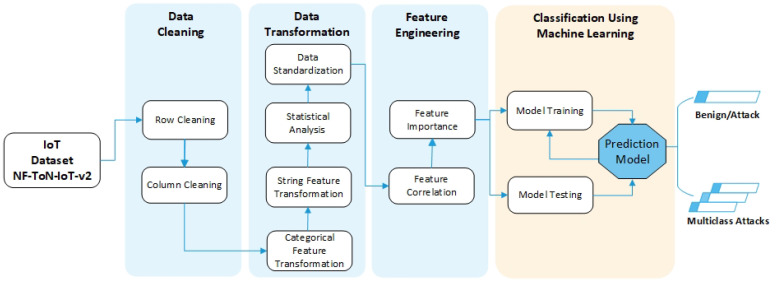
The architecture of the proposed system.

As shown in Figure 1, the proposed system consists of four phases: (1) Data Cleaning, (2) Data Transformation, (3) Feature Engineering, and (4) Classification using Machine Learning.

#### 3.1.1. Data Cleaning

This stage aims to clean and prepare data for analysis by removing incomplete, incorrect, duplicates, and irrelevant rows and columns. Data cleaning is important as it improves the data quality, positively affecting the detection process performance and reducing detection time. As Figure 1 shows, the data cleaning phase consists of two processes:

1. Row cleaning process: deleting any incomplete or noisy rows such as rows with missing values, INF and Null values, and duplicated rows. By the end of this process, 131 rows were dropped from the NF-ToN-IoT-v2 dataset. The remaining rows are complete with no missing values or duplicate rows.
2. Column cleaning: in this process, unnamed columns were excluded. Moreover, as the Label and Attack features are highly correlated, the Label feature was dropped for the Bi-classification experiments, and the Attack feature was deleted for the multi-classification experiments. Features with a single value, such as FTP_COMMAND_RET_CODE, were dropped since they do not contribute to the classification process.

#### 3.1.2. Data Transformation

Data transformation is vital and typical in the pre-processing stage. Data transformation enables the classifier to learn better from the fed dataset. Two types of feature transformation are applied to the dataset: categorical features and string feature transformations.

1. The categorical feature transformation: is obligatory for any classifier model because it can only receive numeric values. For example, the attack feature contains ten classes (nine attack classes and one benign class) that have been encoded into ten numbers from 0 to 9. On the other hand, the binary label feature is encoded into 0 for no-attack (benign) and 1 for the attack (anomaly) class.
2. The String feature transformation is applied to encode a formatted string feature to a numerical one. For example, the features IPV4_SRC_ADDR and IPV4_SRC_ADDR are IP addresses expressed in dotted-quad format (e.g., 192.168.0.1). The IP features cannot effectively contribute to the classification process in this format. Thus, the IPv4Address python class has been applied to convert the string IP format to a number using 256 base conversion.
3. Statistical Analysis: All rows distant from other rows, known as outliers, influence the statistical measure such as mean and deviation. The outliers can be high variance in the dataset due to data extraction or collection issues. The outliers can misrepresent the attack pattern recognition. For this reason, a statistical analysis is applied using the interquartile range (*IQR*) score technique to detect the outliers. *IQR* is a statistical dispersion measure that is equal to the difference between upper and lower quartiles:
  (1) $$\begin{array}{c} IQR=Q3-Q1; \end{array}$$
  where *Q*1 is the first quartile (25th percentile) and *Q*3 is the third quartile (75th percentile. Any row outside the (Q1−1.5×IQR) and (Q3+1.5) range is considered an outlier. Experimentally, when outlier detection is applied to the NF-ToN-IoT-v2 dataset, the number of detected rows as outliers was around 4 million out of 16 million. This meant that a quarter of the dataset would have to be deleted, and consequently, three attack types would be excluded. Therefore, we decided to keep all the dataset rows and apply data scaling to minimize the outliers’ magnitudes in each feature.

#### 3.1.3. Data Standardization

Data standardization is a crucial step in the data preprocessing stage as it improves the performance of any classifier model. As the dataset on hand has been generated from different resources with variant scales for each feature, data standardization is essential to rescale those features. Data standardization eliminates any bias caused by the most significant numeric values negatively affecting the classification process. For this reason, a scaling technique has been applied to the dataset by converting the feature values to set the mean and standard deviation to 0 and 1, respectively. The feature values are standardized as follows:

(2) $$\begin{array}{c} y=\frac{x-mean}{Standard\;Deviation} \end{array}$$

where the mean is calculated as

(3) $$\begin{array}{c} mean=\frac{sum(x)}{count(x)} \end{array}$$

And the Standard Deviation is calculated as

(4) $$\begin{array}{c} Standard\;Deviation=\sqrt{\frac{sum{\left(x-mean\right)}^{2}}{count(x)}} \end{array}$$

### 3.2. Feature Engineering

The section below presents the feature engineering process, including feature correlation and feature importance.

#### Feature Correlation

**Figure 2:**
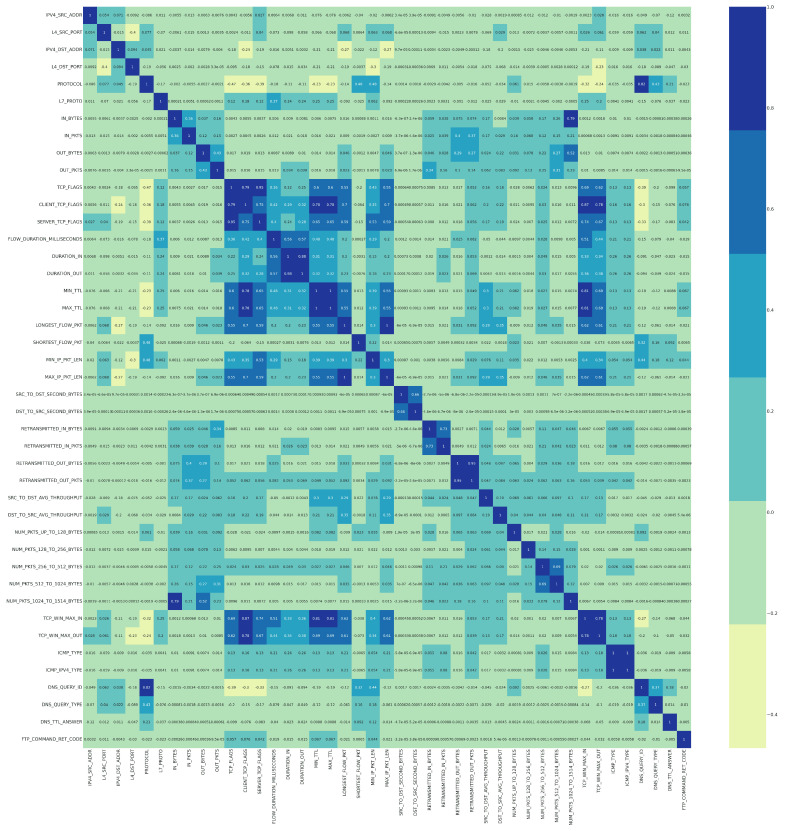
The correlation matrix between the 43 features.

The lower the correlation between the features in the classification process, the better the performance. This is because highly correlated features make similar predictions. Thus, eliminating correlated features reduces the computational time and improves the classifier’s performance. Corr() python function is used to compute the confusion matrix using the Pearson Correlation Coefficient (PCC) [32], defined as

(5) $$\begin{array}{c} r=\frac{\sum \left(x_{i}-\bar{x}\right)\left(y_{i}-\bar{y}\right)}{\sqrt{\sum {\left(x_{i}-\bar{x}\right)}^{2}\sum {\left(y_{i}-\bar{y}\right)}^{2}}} \end{array}$$

where *r* is the correlation coefficient, $x_{i}$ are the values of the x-variable in a sample, $\bar{x}$ is the mean of the values of the x-variable, $y_{i}$ are the values of the y-variable in a sample, and $\bar{y}$ is the mean of the values of the y-variable. Two features are considered positively (or negatively) correlated if the PCC between them is high, near 1 (or – 1). The PCC near 0 means that the features are uncorrelated. Figure 2 shows several correlated features in the dark-colored cells. For example, corr(MIN_TTL, MAX_TTL) = 1, corr(LONGEST_FLOW_PKT, MAX_IP_PKT_LEN) = 1, corr(ICMP_IPV4_TYPE, ICMP_TYPE) = 1, corr(RETRANSMITTE D_OUT_BYTES, RETRANSMITTED_OUT_PKTS) = 0.95, and corr(TCP_FLAGS, SERVER_T CP_FLAGS) = 0.95. A feature importance analysis is applied to decide which of the two correlated features to remove, maintaining the most important feature in the classification process and excluding the other. It is important to emphasize that the feature correlation process has been applied in all experiments with a different set of features extracted via the feature importance process.

## 4. Feature Importance

**Figure 3:**
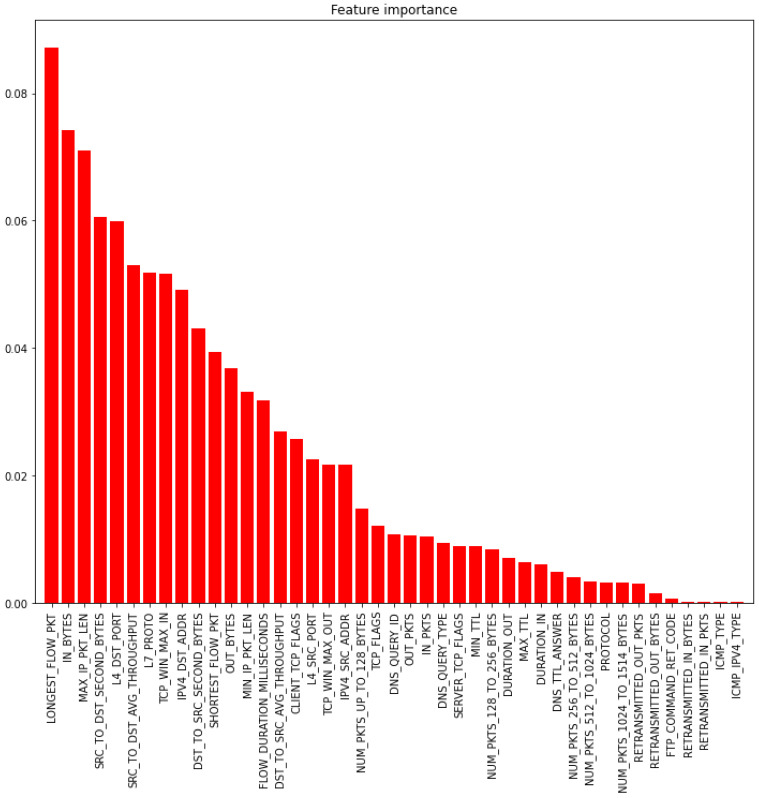
The feature importance for the multi-classification process.

Feature Importance is a method used to determine the contribution of the feature to represent the attack class pattern and then classify it [33]. The better the feature’s contribution in classifying the attack, the higher the importance score. The Random Forest machine has been used in this research work to generate the importance score for each feature. Figure 3 shows the 43 features sorted based on their importance in classifying multi-class attacks.

The number of features to consider when selecting the best features to feed the classifier model is determined based on threshold computation best practice. All features with an importance score greater or equal to the predetermined threshold are maintained as the final feature subset used to train the classifier model. In this research work, the threshold was experimentally determined using several methods, including the ones below:

- Mean: The mean value of the importance scores of the 43 features is 0.02326; this results in a subset consisting of the top 17 features to train the multi-classifier model.
- Median: the median value of the importance scores of the 43 features is 0.01071; this results in a subset consisting of the top 22 features to train the multi-classifier model.
- Through visual analysis of Figure 3, we set the threshold to 0.01; this results in a subset consisting of the top 24 features to train the multi-classifier model.
- Through visual analysis of Figure 3, we set the threshold to 0.02; this results in a subset consisting of the top 19 features to train the multi-classifier model.

**Figure 4:**
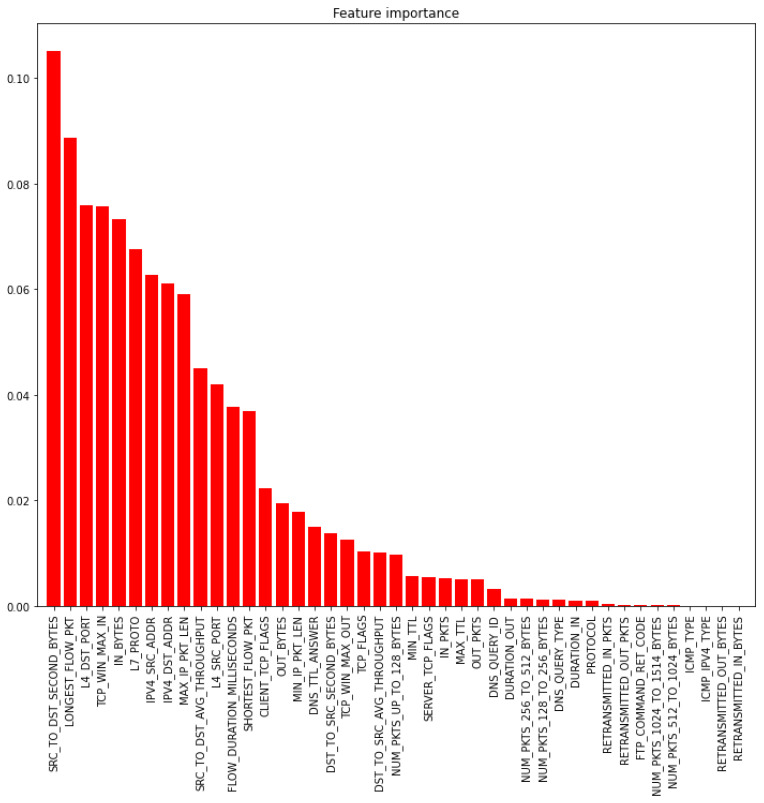
The feature importance for the bi-classification process.

Figure 4 shows the 43 features sorted based on their importance in binary classification (benign vs. attack). As shown in Figure 4, the feature importance score depends on the number of attack classes. The feature importance scores change values because the number of attack classes varies; consequently, the attack pattern representation changes accordingly. It should be noted that the same threshold determination techniques applied in multi-classification are also applied in the bi-classification process.

### 4.1. Classification Using Machine Learning

Several machine learning algorithms were applied to construct a model that can accurately discriminate between ten different classes (nine attacks and a benign). The supervised ML algorithms used in the classification process include Naive Bayes (NB), Random Forest [34] (RF) [35], Decision Tree (DT) [36], and eXtreme Gradient Boosting (XGB) [37].

### 4.2. Performance Evaluation Metrics

**Figure 5:**
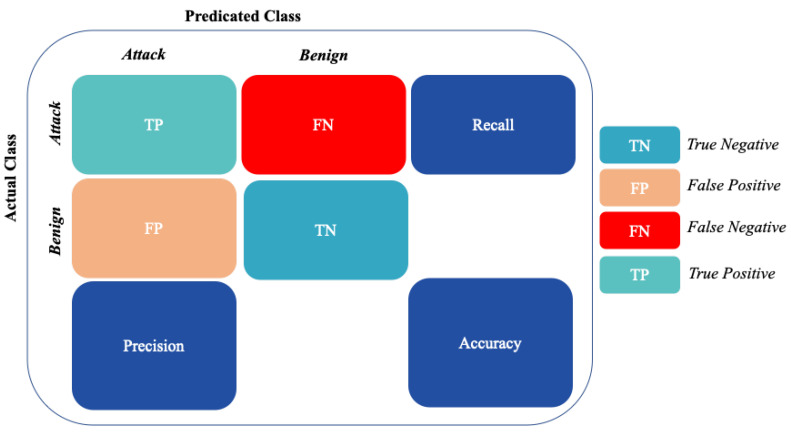
Confusion matrix for NF-ToN-IoT-v2 Bi-classification process.

**Figure 6:**
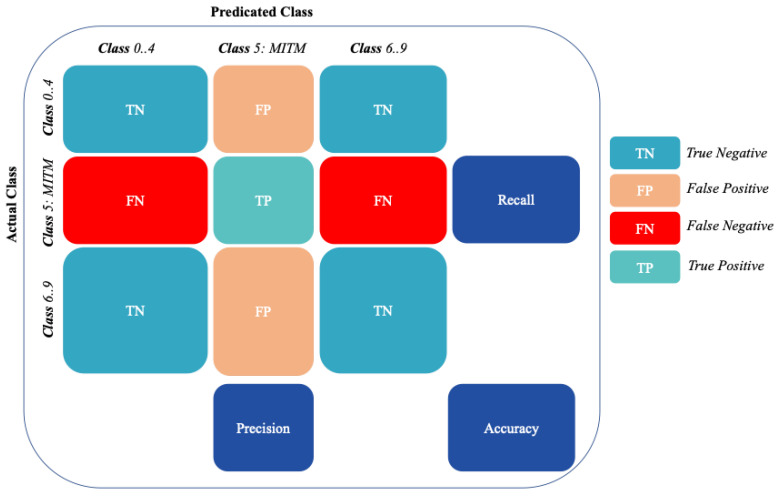
Confusion matrix for NF-ToN-IoT-v2 multi-classification process.

As shown in Figure 5 and Figure 6, Precision, Recall, Accuracy, and F1-score metrics were used to measure the performance of the machine learning models in detecting the benign vs. attack classes in the binary classification and each of the ten classes in the multi-classification.

In the multi-classification process, the Recall, Precision, F1-score, and Accuracy metrics [38] are calculated per class as binary classification (“one-vs-all”). The final metric is the average of all class metrics. As shown in Figure 6, the confusion matrix of classification with ten classes, the True Positive (*TP*), True Negative (*TN*), False Positive (*FP*), and False Negative (*FN*) results, can be obtained for each class k, where 0 ≤ k ≤ n.

The precision measure is the ratio of actual attack records predicted successfully as an attack to the total records predicted as an attack.

(6) $$\begin{array}{c} \mathrm{precision}=\frac{TP}{\left(TP+FP\right)} \end{array}$$

Recall measure is the ratio of actual attack records predicted successfully as an attack to the total records in the attack class.

(7) $$\begin{array}{c} \mathrm{Recall}=\frac{TP}{\left(TP+FN\right)} \end{array}$$

F1-Score is defined as the harmonic mean of Precision and Recall measures.

(8) $$\begin{array}{c} F1-\mathrm{Score}=\frac{2\times (\mathrm{Recall}\times \mathrm{Precision})}{\left(\mathrm{Recall}+\mathrm{Precision}\right)} \end{array}$$

Accuracy measure is the ratio between all correct detection records classes (Attack and No-Attack) and the total number of detection records classes (*TP* + *FP* + *TN* + *FN*).

(9) $$\begin{array}{c} \mathrm{Accuracy}=\frac{\left(TP+TN\right)}{\left(TP+FP+TN+FN\right)} \end{array}$$

## 5. Results and Analysis

This section is divided into two subsections. The first subsection analyzes the best set of features that can adequately represent each attack class. The second subsection evaluates the machine learning classification algorithms in terms of Precision, Recall, F1-score, detection accuracy, and processing time using the NF-ToN-IoT-v2 dataset. We believe the creators may have dropped some of the NetFlow dataset features to eliminate learning bias towards specific sources and destinations. However, the exact number was not explicitly stated in the case of NF-ToN-IoT-v2.

### Feature Selection

Figure 3 indicates the importance of each feature to the RF model used to classify the attack classes. The features are sorted based on their importance, where Longest_Flow_PKT is the most important feature, and ICMP_IP4V_Type is the least important feature for the multi-classification process. In the binary-classification process, in Figure 4, SRC_DST_Secon d_BYTES and RETRANSMITTED_I_BYTES are the most and the least important part, respectively.

To select the best set of features that independently represent each class, several experiments were conducted to determine the threshold and decide which features to choose. Table 3 displays the bi-classification F1-score performance of four data-driven strategies to compute the optimal threshold value. First, through visual analysis of the feature importance, we set the threshold to 0.02, which resulted in 14 features and performance similar to that of all 43 NF features. Then, we tested the performance using the median, mean, and best eight features. Using the mean as a threshold resulted in a small subset of features (13) and high performance.

Table 4 shows the results of the multi-classification performance results using similar thresholds. Using the 17 features above the mean resulted in a high performance similar to that obtained using the entire feature set with less than half of the features.

It is important to note that once the features are selected, we apply a feature filtering process where all features with a correlation coefficient greater than 90% are removed to avoid unnecessary duplication and reduce the number of the selected features.

For the binary-classification process, Table 3 shows that the best threshold for feature selection is the mean = 0.023. This decision is suitable for the accuracy, F1-score, and the number of chosen features. All the features with an importance coefficient greater than the mean are kept while the others are discarded. Consequently, only 13 features are selected for classification, namely: [SRC_TO_DST_SECOND_BYTES, LONGEST_FLOW_PKT, L4_DST_PORT, TCP_WIN_MAX_IN, IN_BYTES, L7_PROTO, IPV4_SRC_ADDR, IPV4_DST_ADDR, MAX_IPvPKT_LEN, SRC_TO_DST_AVG_THROUGHPUT, L4_SRC_PORT, FLOW _DURATION_MILLISECONDS,SHORTEST_FLOW_PKT].

For the multi-classification process, Table 4 shows that the best threshold for feature selection is the mean = 0.023. This decision is suitable for the accuracy, F1-score, and the number of chosen features. All the features with an importance coefficient greater than the mean are kept while the others are discarded. Consequently, only 17 features are selected for classification, namely: [‘LONGEST_FLOW_PKT’, ‘IN_BYTES’, ‘MAX_IP_PKT_LEN’, ‘SRC_TO_DST_SECOND_BYTES’, ‘L4_DST_PORT’, ‘SRC_TO_DST_AVG_THROUGHPUT’, ‘L7_PROTO’, ‘TCP_WIN_MAX_IN’, ‘IPV4_DST_ADDR’, ‘DST_TO_SRC_SECOND_BYTES’, ‘SHORTEST_FLOW_PKT’, ‘OUT_BYTES’, ‘MIN_IP_PKT_LEN’, ‘FLOW_DURATION_MIL LISECONDS’, ‘DST_TO_SRC_AVG_THROUGHPUT’, ‘CLIENT_TCP_FLAGS’, ‘L4_SRC_P ORT’, ‘TCP_WIN_MAX_O’].

Table 5 summarizes the binary-classification results obtained using the most common classification machine learning models: DT, RF, XGB, and NB. The classification was applied using the top 13 features to represent the attack vs. benign class. As can be seen, RF and DT have outperformed XGB and NB models. The results reveal that the selected 13 features are enough for the RF model to flawlessly classify 100% of accuracy rate, all the 4,615,521 records between attack and benign classes.

Table 6 summarizes the multi-classification results obtained using the most common classification machine learning models: DT, RF, XGB, and NB. The classification was applied using the top 17 features to represent the ten different classes. As can be seen, the RF model has outperformed the DT, NB, and XGB models in terms of attack classification. The results reveal that the selected 17 features are enough for the RF model to classify with 98% accuracy all the 4418916 records into either a benign flow or one of the possible nine attacks: Backdoor, Denial of Service (DoS), Distributed Denial of Service (DDoS), Injection, Man in the Middle (MITM), Password, Ransomware, Scanning, Cross-site Scripting (XSS).

**Figure 7:**
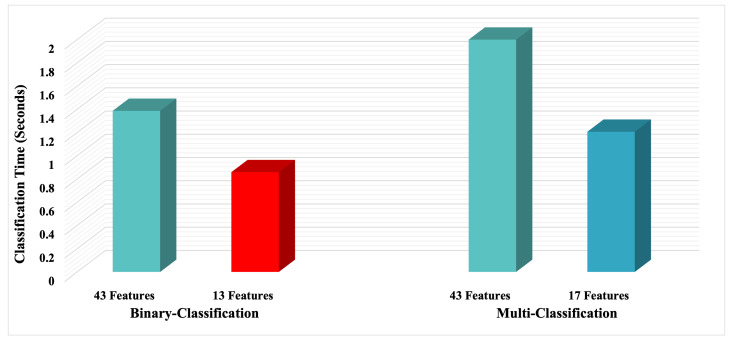
Prediction time for bi-classification and multi-classification using DT model.

Based on the results illustrated in Table 5 and Table 6, and Figure 7, we can see that the 13 selected features are capable of representing the attack vs. benign class with 100% accuracy. Also, the 17 selected features are suitable for independently representing the ten different classes (98% accuracy). Therefore, the low detection rate of the MITM attack (59%) using the Random Forest model is due to the few MITM class records (991 records) compared to the other attack classes represented by thousands of records.

In addition to our attempt to determine the suitable number of NetFlow features for binary and multi-classification, which turned out to be 13 and 17, respectively, we wanted to assess the suitability of the best eight features. In this experiment, we wanted to compare our performance results to those obtained from a previous version of NF-ToN-IoT-v2 (NF-ToN-IoT, 2020) [18]. This dataset consisted of twelve NetFlow features to serve as a standard for other NIDS models’ training and testing. Eight out of the twelve features were utilized in the classification process. The authors achieved an F-1 score of 1.00 and 0.60 in binary and multi-classification, respectively. Our best eight binary classification features achieved a slightly lower F-1 score of 0.97, while the best eight multi-class features resulted in an F-1 score of 0.82, which is significantly higher than 0.60. The authors understand that the dataset creators decided to drop certain features from the twelve. Still, we were interested in comparing the results acquired using eight features on each side.

All experiments’ results were carried out using Google Colab platform. Figure 7 shows the difference in testing time when using all the features compared to the subsets utilized. A smaller subset will result in a faster processing time, making the system more suitable for real-time detection.

Table 7 shows that the attack classification performance is similar to the state of art method using only 13 and 17 instead of 43 features. In other words, using only 30% (Dimensionality Reduction rate = 70%) and 40% (Dimensionality Reduction rate = 60%) of the number of features were enough to represent the two and ten classes, respectively.

## 6. Conclusions

Anomaly-based detection models are trained to determine normal and flag suspicious behaviors. Networks contain many features that can be used to train IDS. For example, NetFlow, a networking industry-standard protocol, provides a rich set of fields (over 70 in version 9) that can be used for that purpose [39]. A recent study proposed a standard set of 43 NetFlow features that resulted in high accuracy [14]. Netflow is a Cisco standard that provides a huge amount of network data that happened to be outstanding when it comes to malicious attack detection. The 2021 study included the creation of a massive NetFlow-based dataset (NF-ToN-IoT-v2) with over 16 million records. Our goal was different as we wanted to utilize this new dataset and further reduce the number of features while maintaining high accuracy results. A lower number of features means faster processing time, which is essential in deploying an anomaly-based detection system.

During our work, we examined several sets of features. We concluded that NetFlow features with importance values above the mean of all feature values are sufficient to predict malicious behavior. Indeed, 13 features were enough to perform binary classification with an F1-score of 1.00, and 17 features were adequate to achieve an F1-score of 0.98 in the case of multi-classification. These results are identical to those obtained using the 43 features [14]. Furthermore, our approach reduced the prediction time by 38% and 40% in binary and multi-classification, respectively.

Our study tested the performance using DT, RF, XGB, and NB. The results achieved by DT and RF were the highest. In our future work, we plan to assess the suitability of this threshold on other NetFlow-based datasets.

## Figures and Tables

**Table 1:** Breakdown of the dataset classes.

Count	Class
Benign	6,099,469
Backdoor	16,809
Denial of Service (DoS)	712,609
Distributed Denial of Service (DDoS)	2,026,234
Injection	684,465
Man in the Middle (MITM)	1,153,323
Password	16,809
Ransomware	3425
Scanning	3,781,419
Cross-site Scripting (XSS)	2,455,020

**Table 2:** NF-ToN-IoT-v2 features.

Feature	Description
IPV4_SRC_ADDR	IPv4 source address
IPV4_DST_ADDR	IPv4 destination address
L4_SRC_PORT	IPv4 source port number
L4_DST_PORT	IPv4 destination port number
PROTOCOL	IP protocol identifier byte
L7_PROTO	Layer 7 protocol (numeric)
IN_BYTES	Incoming number of bytes
OUT_BYTES	Outgoing number of bytes
IN_PKTS	Incoming number of packets
OUT_PKTS	Outgoing number of packets
FLOW_DURATION_MILLISECONDS	Flow duration in milliseconds
TCP_FLAGS	Cumulative of all TCP flags
CLIENT_TCP_FLAGS	Cumulative of all client TCP flags
SERVER_TCP_FLAGS	Cumulative of all server TCP flags
DURATION_IN Client	to Server stream duration (msec)
DURATION_OUT	Client to Server stream duration (msec)
MIN_TTL	Min flow TTL
MAX_TTL	Max flow TTL
LONGEST_FLOW_PKT	Longest packet (bytes) of the flow
SHORTEST_FLOW_PKT	Shortest packet (bytes) of the flow
MIN_IP_PKT_LEN	Len of the smallest flow IP packet observed
MAX_IP_PKT_LEN	Len of the largest flow IP packet observed
SRC_TO_DST_SECOND_BYTES	Src to dst Bytes/sec
DST_TO_SRC_SECOND_BYTES	Dst to src Bytes/sec
RETRANSMITTED_IN_BYTES	Number of retransmitted TCP flow bytes (src->dst)
RETRANSMITTED_IN_PKTS	Number of retransmitted TCP flow packets (src->dst)
RETRANSMITTED_OUT_BYTES	Number of retransmitted TCP flow bytes (dst->src)
RETRANSMITTED_OUT_PKTS	Number of retransmitted TCP flow packets (dst->src)
SRC_TO_DST_AVG_THROUGHPUT	Src to dst average thpt (bps)
DST_TO_SRC_AVG_THROUGHPUT	Dst to src average thpt (bps)
NUM_PKTS_UP_TO_128_BYTES	Packets whose IP size ≤ 128
NUM_PKTS_128_TO_256_BYTES	Packets whose IP size > 128 and ≤256
NUM_PKTS_256_TO_512_BYTES	Packets whose IP size > 256 and ≤512
NUM_PKTS_512_TO_1024_BYTES	Packets whose IP size > 512 and ≤1024
NUM_PKTS_1024_TO_1514_BYTES	Packets whose IP size > 1024 and ≤1514
TCP_WIN_MAX_IN	Max TCP Window (src->dst)
TCP_WIN_MAX_OUT	Max TCP Window (dst->src)
ICMP_TYPE	ICMP Type × 256 + ICMP code
ICMP_IPV4_TYPE	ICMP Type
DNS_QUERY_ID	DNS query transaction Id
DNS_QUERY_TYPE	DNS query type (e.g., 1 = A, 2 = NS.)
DNS_TTL_ANSWER	TTL of the first A record (if any)
FTP_COMMAND_RET_CODE	FTP client command return code

**Table 3:** Feature importance performance for binary-classification Using DT.

Class	All Features	Importance ≥ 0.02	Importance ≥ Median (0.0096)	Importance ≥ Mean (0.0232)	Best 8 Features
	F1-score	F1-score	F1-score	F1-score	F1-score
0. Benign	0.99	0.99	0.99	0.99	0.96
1. Attack	1.00	1.00	1.00	1.00	0.98
Weighted avg	1.00	1.00	1.00	1.00	0.97
Final # of features used	43 (none removed)	Top 14 features ${}^{1}$	Top 22 features ${}^{2}$	Top 13 features ${}^{3}$	Top 8 features ${}^{4}$

^1^ from SRC_TO_DST_SECOND_BYTES to CLIENT_TCP_FLAGS; ^2^ from SRC_TO_DST_SECOND_BYTES to NUM_PKTS_UP_TO_128_BYTES; ^3^ from SRC_TO_DST_SECOND_BYTES to SHORTEST_FLOW_PKT—Adopted approach; ^4^ from SRC_TO_DST_SECOND_BYTES to IP4_DST_ADDR.

**Table 4:** Feature importance performance for multi-classification Using DT.

Class	All Features	Importance ≥ 0.02	Importance ≥ 0.01	Importance ≥ Median (0.0107)	Importance ≥ Mean (0.232)
2-6	F1-score	F1-score	F1-score	F1-score	F1-score
0. Benign	0.99	1.00	0.99	0.99	0.99
1. Backdoor	1.00	1.00	1.00	1.00	1.00
2. DoS	0.99	0.99	0.99	0.99	0.98
3. DDoS	0.89	0.80	0.89	0.89	0.77
4. Injection	0.91	0.92	0.91	0.91	0.91
5. MITM	0.55	0.60	0.55	0.55	0.58
6. Password	0.97	0.97	0.97	0.97	0.97
7. Ransomware	0.97	0.98	0.97	0.98	0.98
8. Scanning	1.00	1.00	1.00	1.00	1.00
9. XSS	0.95	0.94	0.95	0.95	0.93
Weighted avg	0.98	0.98	0.98	0.98	0.98
Final # of features used	43 (none removed)	Top 18 features	Top 23 features	Top 22 features	Top 17 features

**Table 5:** Binary classification using features with importance ≥ mean (top 13 features).

	DT			RF			XGB			NB		
Class	PR	RC	F1-Score	PR	RC	F1-Score	PR	RC	F1-Score	PR	RC	F1-Score
0. Benign	1.00	0.99	0.99	1.00	0.99	1.00	0.98	0.97	0.97	0.93	0.04	0.07
1. Attack	1.00	1.00	1.00	1.00	1.00	1.00	0.98	0.99	0.98	0.62	1.00	0.76
Weighted avg	1.00	1.00	1.00	1.00	1.00	1.00	0.98	0.98	0.98	0.74	0.62	0.50
Accuracy	1.00			1.00			0.98			0.62		

**Table 6:** Multi-classification using features with importance ≥ mean (top 17 features).

	DT			RF			XGB			NB		
Class	PR	RC	F1-Score	PR	RC	F1-Score	PR	RC	F1-Score	PR	RC	F1-Score
0. Benign	0.99	1.00	0.99	1.00	1.00	1.00	0.96	0.98	0.97	0.98	0.01	0.03
1. Backdoor	1.00	1.00	1.00	1.00	1.00	1.00	1.00	0.99	0.99	1.00	0.97	0.99
2. DoS	0.98	0.98	0.98	0.98	0.98	0.98	0.93	0.97	0.95	0.85	0.48	0.61
3. DDoS	0.77	0.78	0.77	0.78	0.78	0.78	0.85	0.81	0.83	0.42	0.56	0.48
4. Injection	0.91	0.91	0.91	0.93	0.91	0.92	0.85	0.64	0.73	0.32	0.21	0.25
5. MITM	0.58	0.58	0.58	0.59	0.59	0.59	0.94	0.44	0.60	0.06	0.00	0.01
6. Password	0.97	0.97	0.97	0.97	0.97	0.97	0.87	0.89	0.88	0.47	0.80	0.59
7. Ransomware	0.99	0.98	0.98	0.99	0.99	0.99	0.95	0.78	0.85	0.00	0.16	0.01
8. Scanning	1.00	1.00	1.00	1.00	1.00	1.00	0.97	0.95	0.96	0.39	0.98	0.56
9. XSS	0.94	0.93	0.93	0.93	0.95	0.94	0.88	0.94	0.91	0.61	0.72	0.66
Weighted avg	0.98	0.98	0.98	0.98	0.98	0.98	0.94	0.94	0.94	0.71	0.45	0.35
Accuracy	0.98			0.98			0.94			0.45		

**Table 7:** Comparison between the proposed method performance and state-of-the-art method using NF-ToN-IoT-v2.

Output Class	Measure	Sarhan et al. [14]	Proposed Method
	Feature Set	43	13
Binary Classification Begin vs. Attack	Accuracy	99.64%	100%
	F1-score	1.00	1.00
	Dimensionality Reduction rate	-	70%
	Feature Set	43	17
Multi-Classification Classes 0-9	Accuracy	98.05%	98%
	F1-score	0.98	0.98
	Dimensionality Reduction rate	-	60%
